# Dual Action of Ivy and Strawberry Essential Oils: Induction of *MdPR10* Gene Expression and Antimicrobial Effects in Apple Fruits

**DOI:** 10.3390/ijms27010311

**Published:** 2025-12-27

**Authors:** Lucia Urbanová, Jana Žiarovská, Stefania Garzoli, Soham Bhattacharya, Miroslava Kačániová, Maciej Ireneusz Kluz

**Affiliations:** 1 Research Centre AgroBioTech, Slovak University of Agriculture, Trieda Andreja Hlinku 2, 94976 Nitra, Slovakia; lucia.urbanova@uniag.sk; 2 Institute of Plant and Environmental Sciences, Faculty of Agrobiology and Food Resources, Slovak University of Agriculture, 94976 Nitra, Slovakia; 3 Department of Chemistry and Technologies of Drug, Sapienza University, 00185 Rome, Italy; stefania.garzoli@uniroma1.it; 4 Department of Agroecology and Crop Production, Faculty of Agrobiology, Food and Natural Resources, Czech University of Life Sciences Prague, 165 00 Suchdol, Czech Republic; bhattacharya@af.czu.cz; 5 Institute of Horticulture, Faculty of Horticulture and Landscape Engineering, Slovak University of Agriculture, 94976 Nitra, Slovakia; miroslava.kacaniova@gmail.com; 6 School of Medical & Health Sciences, VIZJA University, 01043 Warszawa, Poland; 7 Andrzej Frycz Modrzewski Krakow University, 30-705 Kraków, Poland; mkluz@uafm.edu.pl

**Keywords:** *MdPR10* gene expression, secondary metabolites, *Malus domestica* Borkh., *Xanthomonas arboricola*, *Pectobacterium carotovorum*, *Pseudomonas syringae*, *Agrobacterium radiobacter*, *Priestia megaterium*

## Abstract

One significant trend in the research of plant treatment methods is that regarding the use of natural-based methods in plant protection. In this study, antimicrobial activity and changes in *MdPR10* gene expression were tested for a total of five plant pathogens in a model of apple fruits, where strawberry and ivy EOs were used. The vapor-phase chemical composition of both EOs was profiled using HS-GC-MS. qRT-PCR was applied for a bacterial response analysis, together with disk diffusion assays, and minimum inhibitory concentrations were determined. To elucidate the molecular basis of the antibacterial potential of essential oils (EOs), docking analyses were performed. For *Xanthomonas arboricola* and *Pectobacterium carotovorum*, the presence of EOs resulted in the downregulation of *MdPR10*. Strawberry EO was more effective against weakly virulent strains of bacteria; ivy EO had greater inhibitory effects. HS-GC-MS detected 13 volatiles in strawberry EO—dominated by ethyl butyrate, ethyl 2-methylbutanoate, ethyl hexanoate, and ethyl 3-methylbutanoate—and 16 in ivy EO, characterized by monoterpenes and monoterpenoids with 1,8-cineole as the principal component. *P*-cymene showed the most potent binding activity against D-alanine–D-alanine ligase. Ivy EO has the potential to be effective as a natural preservative alternative mainly in postharvest technology.

## 1. Introduction

The use of synthetic pesticides in agriculture has been widespread since the 1940s. After decades of intensive application, concerns have emerged about their direct toxic effects on the environment and human health [1], prompting efforts to find environmentally safer alternatives. The adoption of greener chemical solutions in agriculture aligns with the visions of the European Union [2]. One promising alternative is the use of biopesticides—substances derived from living organisms or certain minerals that protect plants against pests.

EOs are classified as a subgroup of botanical biopesticides [3]. Their defense mechanisms, encoded in the specific plant genome, can be used as concentrations to protect other plant species. The importance of EOs lies in their target specificity, potential for broad-spectrum application (due to their complex chemical composition), high efficacy, and environmental safety. In organic agriculture, the degradability of insecticidal formulations is one of the critical factors, and EOs perform well in this regard [4]. Although some purified components of EOs may exhibit a slight toxicity to mammals, they are generally considered non-toxic to mammals, fish, and birds, with few exceptions [5]. EOs are primarily used as repellents and insecticides (e.g., *Mentha piperita* L.—Lamiaceae) or for the control of fungal pathogens (e.g., *Pelargonium roseum* Ehrh.—Geraniaceae, *Lavandula angustifolia* Mill.—Lamiaceae, *Juniperus virginiana* L.—Cupressaceae). Oils from eucalyptus and lemongrass have also demonstrated antimicrobial properties [6,7]. The use of biopesticides is primarily limited by their own production, which, if scaled for widespread commercial use, could compete with the cultivation of food crops. Additionally, the extraction of specific components may pose environmental concerns when harmful solvents are employed [4]. The application of EOs also raises concerns regarding the safety of treated crops for individuals with acute allergic responses.

Several EO components exhibit both antioxidant and antimicrobial effects, often attributed to the same chemical compounds. The antioxidant properties help protect plants by neutralizing free radicals, while their chemical structures also enable interactions with microbial enzymes, the inhibition of bacterial growth, or the disruption of bacterial cell walls. Compounds such as polyphenols, flavonoids, and terpenes perform both functions [8]. For example, phytosterols predominantly have antioxidant effects [9], while other components, such as tocopherols, act as signaling molecules in the plant stress response [10].

*Malus domestica* Borkh. is mostly cultivated in temperate climates, and its fruits are relevant in the food industry, consumed not only fresh but also as an ingredient in processed food. Modern cultivars are bred for resistance to their most significant diseases, including apple scabs and powdery mildew. Smeralda is a highly disease-resistant apple variety that appeared in 2011 in Italy. It is suitable for fresh consumption due to its favorable taste characteristics. Despite its higher water content, it withstands long-term storage [11]. The resistance to pathogens in apples is mediated by a complex network of metabolic pathways, including pathogenesis-related (PR) proteins. PR-10 proteins are ubiquitous and homologously coded by *ypr10* genes, playing a key role in plant defense by degrading the RNA of invading pathogens [12].

Plant-pathogenic bacteria are a major source of biotic stress, leading to reduced growth, vitality, and productivity in crops. *Xanthomonas arboricola*, particularly its pathovar *fragariae*, is a well-known pathogen of fruit trees and is currently listed as a regulated pest within the EU [13,14,15]. Soft root caused by *Pectobacterium* spp. poses serious challenges in potato production, especially under humid conditions. The lack of effective chemical treatments and resistant cultivars makes this disease difficult to control [16,17]. *Pseudomonas syringae* affects a wide range of economically important plants. It enters host tissues through natural openings and continues to be a global threat due to its high genetic variability and adaptability [18,19]. Although not pathogenic in the classical sense, *Agrobacterium radiobacter* and *Priestia megaterium* play an important role in the soil environment. These bacteria can improve plant health and influence microbial balance in the rhizosphere, potentially suppressing harmful microorganisms [20,21]. With increasing restrictions on synthetic pesticides, plant EOs are being explored as a natural and sustainable approach to mitigating plant stress and managing bacterial diseases under both laboratory and real-life conditions.

One of the aims of this study was to investigate whether EOs from ivy (*Hedera helix* L.) and strawberries (*Fragaria* × *ananassa*) could influence the expression of the plant defense gene *MdPR10* in apple fruits (*Malus domestica* Borkh.) under bacterial stress. This gene encodes Mal d 1 (PR-10), which is not only involved in the plant’s immune response but is also known as a major allergen in sensitive individuals. By monitoring gene expression, we aimed to explore whether EOs can trigger natural plant defenses and potentially affect the levels of allergenic protein in the fruit. Along with this, we evaluated the antimicrobial activity of these EOs against selected phytopathogenic bacteria, both in vitro and in situ, to determine their dual role—the direct inhibition of bacterial growth and the stimulation of defense-related responses in the plant.

To our knowledge, this is the first study concerning gene expression, antimicrobial properties, molecular docking, and the possible impact of EOs on plant allergen levels. This integrative approach combining gene expression analysis and antimicrobial assays offers new insights into the potential dual role of EOs in sustainable agriculture.

## 2. Results

### 2.1. Vapor-Phase Chemical Composition of EOs

HS-GC-MS analysis allowed us to detect and identify 13 volatile components in strawberry EO and 16 in ivy EO. Specifically, strawberry EO was rich in fatty acid esters, with ethylbutyrate (32.2%), ethyl 2-methylbutanoate (30.6%), ethyl hexanoate (12.3%), and ethyl-3-methylbutanoate (11.6%) as the most abundant (Table 1). The chemical volatile profile of ivy EO was characterized by monoterpenoids (48.7%) and monoterpenes (50.0%). Among monoterpenoids, 1,8-cineole (47.5%) was the principal component.

### 2.2. Mal d PR10 Expression Changes

The PCR efficiencies in the expression analysis were 101.09% (*MdACT*) and 108.22% (*MdPR10*), with the coefficients of determination of R^2^ = 0.997 (*MdACT*) and R^2^ = 0.998 (*MdPR10*). This is a result of the presence of inhibitors in samples with the highest proportion of bacteria.

**Figure 1:**
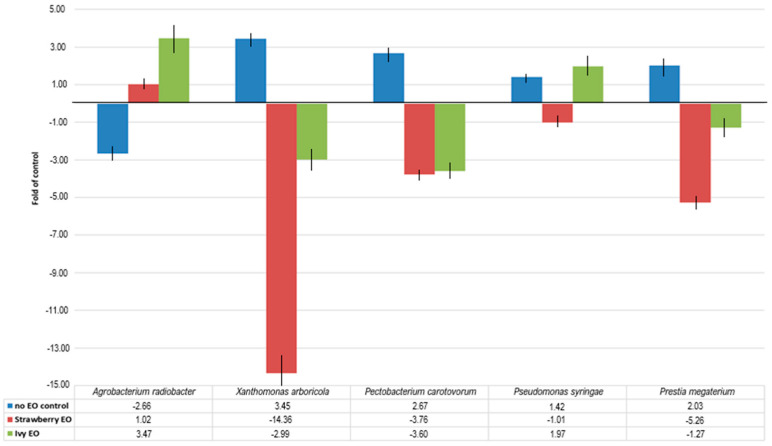
Expression profiles of *MdPR10* gene in different pathogen and EO variants. Y axis expression changes compared to control variants, with actin as internal reference (NCBI ID DT002474).

The response of the expression of *MdPR10* in apple fruits to the presence of each bacterial species varied. Statistically significant changes in *MdPR10* expression were observed in response to three out of five bacterial treatments: *Agrobacterium radiobacter*, *Xanthomonas arboricola*, and *Pectobacterium carotovorum* ((*p* < 0.05)/Figure 1).

An exposure to *A. radiobacter* resulted in a negative fold change of −2.66, suggesting that this species may not strongly induce *MdPR10* expression in apple fruits, or that it interacts with the host through a pathway independent of the *MdPR10*-mediated stress response.

The highest expression was induced by *X. arboricola*, with a nearly 3.5-fold increase, followed by *P. carotovorum*, which caused a 2.67-fold change, both compared to the 7th-day control. The application of strawberry EO with *X. arboricola* in apple fruits caused the most significant change in the treatment collection, resulting in more than a 14-fold decrease compared to the no-EO control (*p* < 0.001).

By contrast, the slight increase in *MdPR10* expression caused by *Priestia megaterium* was not statistically significant, and the addition of strawberry EO reduced *MdPR10* expression to levels below those observed in the no-EO control (*p* < 0.001).

The presence of *P. carotovorum* led to a 2.67-fold increase in *MdPR10* expression. Similarly to the previous two bacteria, the application of strawberry EO significantly reduced *MdPR10* activity below the no-EO control (*p* < 0.01).

In samples treated with *A. radiobacter* and strawberry EO, *MdPR10* expression levels remained nearly unchanged compared to the corresponding no-EO control.

*Pseudomonas syringae* did not induce statistically significant changes in *MdPR10* expression, either in the presence or absence of strawberry EO (*p* > 0.05). Overall, ivy EO proved to be less effective in suppressing the apple stress response to the tested bacteria compared to strawberry EO. When ivy EO was applied with *A. radiobacter*, the stress response increased to nearly 3.5-fold compared to the no-EO control (*p* < 0.01). In contrast, compared to the 7th-day control, the expression increased statistically insignificantly, which may indicate that ivy EO itself causes a stress response in apple fruits. Contrasting with the previous combination, the stress response increased in the presence of *P. syringae*, with a nearly 2-fold change (*p* < 0.05), and was nearly 2.8-fold higher compared to the 7th-day control. Thus, the combination of both factors induces *MdPR10* expression even more than the bacteria itself. The application of ivy EO on *P. megaterium* showed a statistically insignificant change in the expression of *MdPR10* (*p* > 0.05). The fold change for strawberry and ivy EOs was very similar in combination with *P. carotovorum*, with a high significance (*p* < 0.01). In the case of the bacteria *X. arboricola*, the expression was only three times lower than without the application of ivy EO (*p* > 0.05).

### 2.3. Antimicrobial Activity In Vitro

The results of the in vitro antimicrobial testing of strawberry EO indicate a mild to moderate efficacy against the tested phytopathogenic bacteria (Table 2). The highest sensitivity was observed in *P. megaterium*, which exhibited the largest inhibition zone (7.33 mm) and the lowest MIC_50_ (3.37 mg/mL) and MIC_90_ (3.51 mg/mL) values (Table 3).

A relatively good efficacy was also observed for *A. radiobacter* and *X. arboricola*, with inhibition zones of 5.33 mm and 5.67 mm, respectively, although their MIC values were slightly higher than those recorded for *P. megaterium*.

Conversely, the lowest efficacy was observed against *P. carotovorum* and especially *P. syringae*. For *P. syringae*, the smallest inhibition zone was recorded (2.33 mm), along with the highest MIC_90_ value of 7.01 mg/mL.

### 2.4. In Situ Antimicrobial Activity of Strawberry Essential Oil

The results demonstrate that strawberry EO, when applied in the vapor phase to apple slices, exerts a distinct dose-dependent antimicrobial effect on all tested bacterial species (Table 4). At the highest concentration (500 µg/L), surface growth inhibition exceeded 45% for *X. arboricola*, *P. carotovorum*, *P. megaterium*, and *P. syringae*. This indicates that the EO vapors maintained a sufficient partial pressure in the micro-atmosphere to exert broad-spectrum antimicrobial effects.

### 2.5. In Vitro Effect of Ivy Essential Oil

The in vitro results confirmed that ivy EO exhibited antimicrobial activity against all tested bacterial strains, though the extent of inhibition varied by species (Table 5). The largest inhibition zone was recorded for *A. radiobacter* (11.67 mm), despite this species also showing the highest MIC_90_ (7.52 mg/mL) (Table 6). This apparent discrepancy—large inhibition zones but high MIC values—may reflect the different sensitivities of agar diffusion and broth dilution methods, which are influenced by compound diffusion rates and solubility [22]. The lowest MIC_50_ value was observed for *P. megaterium* (4.73 mg/mL), which also showed a relatively large inhibition zone (8.33 mm).

### 2.6. In Situ Antimicrobial Activity of Ivy Essential Oil

The in situ evaluation of ivy EO revealed a strong concentration-dependent inhibitory effect against all tested bacterial strains (Table 7). At the highest concentration (500 µg/L), the surface growth of all pathogens was reduced by over 84%, with *A. radiobacter*, *P. megaterium*, and *P. carotovorum* showing the most pronounced responses. Notably, even the most resistant strain at lower concentrations, *P. syringae*, was substantially inhibited (86.89%) at 500 µg/L. This confirms the broad-spectrum effectiveness of ivy EO when applied as a vapor-phase treatment.

### 2.7. In Silico Molecular Docking

The molecular interactions between the bioactive constituents of strawberry and ivy EOs and the selected bacterial target enzymes were evaluated through a molecular docking analysis. The calculated binding affinities of all ligand–protein complexes are summarized in Table 8. Compounds identified from strawberry EOs exhibited low to moderate binding affinities toward the selected proteins, with docking scores ranging from −3.8 to −6.1 kcal mol^−1^. In contrast, the major constituents of ivy EOs demonstrated comparatively stronger interactions, with binding energies between −4.1 and −7.9 kcal mol^−1^. 

**Figure 2:**
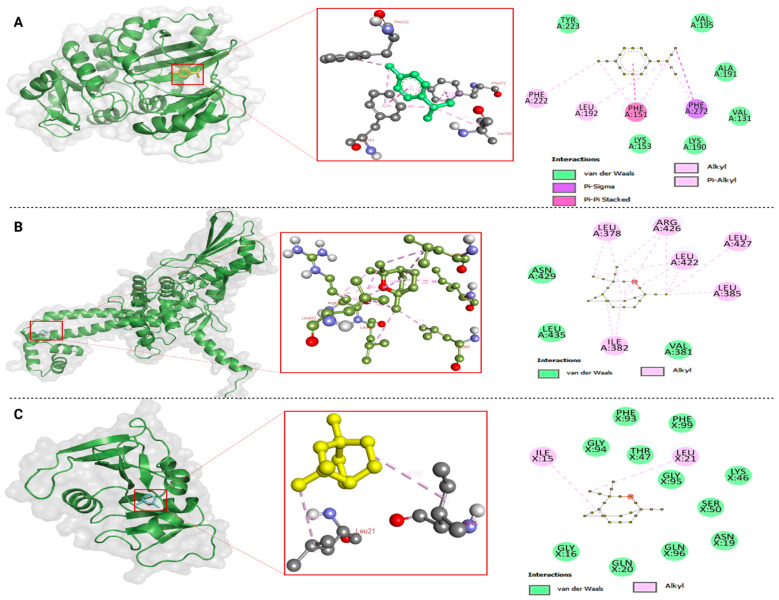
Three-dimensional and two-dimensional binding interactions of the best docked complex (**A**) 2ZDQ with p-cymene, (**B**) 3RAE with 1,8-cineole, and (**C**) 3SRW with 1,8-cineole.

Among these, *p*-cymene showed the most potent binding activity (−7.9 kcal mol^−1^) against D-alanine–D-alanine ligase, primarily mediated by hydrophobic interactions, including π–π stacking with PHE151 (chain A), π–σ bonding with PHE272 (chain A), and π–alkyl interactions with PHE222 and LEU192 (chain A) (Figure 2A). Furthermore, *p*-cymene also displayed the most favorable affinities toward other essential bacterial enzymes, including DNA gyrase (−5.8 kcal mol^−1^), dihydropteroate synthase (−5.1 kcal mol^−1^), and penicillin-binding protein 1a (−6.0 kcal mol^−1^).

Likewise, 1,8-cineole, the predominant constituent of ivy EO, exhibited a strong binding affinity for topoisomerase IV (−6.1 kcal mol^−1^) through hydrophobic alkyl interactions involving the A chains of LEU378, ILE382, LEU385, LEU422, ARG426, and LEU427 (Figure 2B). It also formed stable interactions with dihydrofolate reductase (−6.0 kcal mol^−1^), primarily through alkyl contacts with ILE15 and LEU21 (chain A) (Figure 2C).

## 3. Discussion

EOs can directly influence gene expression in plants, both by priming defense pathways and regulating biosynthetic enzymes. Their effects are context-dependent, varying with developmental stage, plant species/cultivar, and the specific EO components used [22]. EO treatments have been reported to upregulate plant defense-related genes, including those encoding chitinase and thaumatin-like proteins in eggplant, contributing to an increased resistance against pests or pathogens [23]. Here, the *ypr10* gene was evaluated for changes in expression patterns under the biotic stress of different pathogens and application of two types of EOs. Previously, studies on wild blueberry phenotypes have demonstrated varied expression patterns of genes’ PR proteins in response to *B. cinerea* infections where they were highly expressed, with the gene of PR4 peaking at 12 h post-inoculation [24].

The composition of the vapor phase of EOs was reflected in their effects: strawberry EO was dominated by fatty-acid ethyl esters (ethyl butyrate, ethyl 2-methylbutanoate, ethyl hexanoate, ethyl 3-methylbutanoate), in agreement with the literature on dominant strawberry headspace aromatics in fruits and products [25,26]. By contrast, ivy EO was rich in monoterpenes/monoterpenoids, with a high share of 1,8-cineole, whose antimicrobial activity—including effects on both Gram-negative and Gram-positive bacteria via membrane disruption—is well documented, supporting the stronger inhibitory effects observed for ivy [27]. The variability in EO chemical profiles by origin, plant part, and extraction method is known and may explain inter-study differences. At the host level, the downregulation of *MdPR10* upon EO exposure suggests a differential modulation of defense pathways; the role of PR-10 proteins in apple resistance and their interactions with pathogen effectors confirm the functional relevance of *MdPR10* in host–pathogen physiology [28]. Together, these results support ivy EO as a natural postharvest alternative, with successful applications depending on chemotype standardization and dosing.

An exposure to *A. radiobacter* neither strongly induces *MdPR10* expression in apple fruits nor interacts with the host through a pathway independent of the *MdPR10*-mediated stress response. The microbiological nature of *A. radiobacter* is known to inhabit the rhizosphere and is not typically associated with fruit infection. Its ecological role is more connected to the root environment, where it may interact with the plant differently than pathogens that infect fruit tissue [21]. Therefore, its presence may not trigger a typical defense reaction in apple fruits. The highest expression of *MdPR10* was induced by *X. arboricola*, with a nearly 3.5-fold increase, followed by *P. carotovorum*, which caused a 2.67-fold change. *X. arboricola* is a known phytopathogen of fruit trees, capable of directly infecting fruit tissue and inducing a strong biotic stress response in the host [14]. This explains its marked effect on the expression of *MdPR10*, especially when combined with the presence of EO, which may further modulate the host response through an interaction with stress signaling pathways. By contrast, the slight increase in *MdPR10* expression caused by *Priestia megaterium* was not statistically significant. *P. megaterium* is generally considered a plant growth-promoting bacterium (PGPR), commonly associated with the rhizosphere [18]. Its interaction with apple fruit tissue is likely weak or non-pathogenic, which may explain the absence of a typical defense response. The presence of *P. carotovorum* led to a 2.67-fold increase in *MdPR10* expression. Similarly to the previous two bacteria, the application of strawberry EO significantly reduced *MdPR10* activity below the no-EO control. *P. carotovorum* is a soft rot pathogen that produces pectolytic enzymes and actively invades plant parenchyma, including fruit tissues [16]. Its interaction with apple fruits likely activates defense genes such as *MdPR10*, while EO treatment may interfere with this response or reduce bacterial virulence. In samples treated with *A. radiobacter*, *MdPR10* expression levels remained nearly unchanged compared to the corresponding no-EO control. *A. radiobacter* primarily colonizes the rhizosphere and is not typically involved in fruit tissue interactions, which may explain its limited ability to affect gene expression in the fruit.

The results of the in vitro antimicrobial testing of strawberry EO indicate a mild to moderate efficacy against the tested phytopathogenic bacteria, and the highest sensitivity was observed in *P. megaterium*, which exhibited the largest inhibition zone (7.33 mm) and the lowest MIC_50_ (3.37 mg/mL) and MIC_90_ (3.51 mg/mL) values. This bacterium is known for its saprophytic and often probiotic properties, and, under in vitro conditions, it is typically less resistant to plant-derived EOs [29,30]. *A. radiobacter*, as a rhizosphere-associated strain, is not a typical fruit pathogen, which may explain its greater susceptibility to the volatile compounds of strawberry EO. *X. arboricola* is a significant pathogen of fruit trees, and its sensitivity to EOs may be influenced by the specific chemical composition of the EO, particularly the presence of phenylpropanoids and linalool, which have previously been shown to be effective against the genus *Xanthomonas* [30,31]. Conversely, the lowest efficacy was observed against *P. carotovorum* and especially *P. syringae*. For *P. syringae*, the smallest inhibition zone was recorded (2.33 mm), along with the highest MIC_90_ value of 7.01 mg/mL. These findings confirm the high tolerance of the *Pseudomonas* genus to phytochemicals, which may be attributed to its ability to form biofilms, express efficient efflux pumps, or produce enzymes capable of degrading volatile antimicrobial compounds [29,30].

The previous literature confirms that EOs can be highly effective in the vapor phase due to the volatile nature of their terpenoids and phenolic compounds, which destroy cell membranes and disrupt bacterial homeostasis [32,33]. Intermediate concentrations (250 µg/L) still maintained a strong activity (~32%), particularly against phytopathogens (*X. arboricola*, *P. carotovorum*). However, bactericidal effects diminished sharply at lower doses (125 and 62.5 µg/L), underscoring the importance of adequate dosing to achieve an effective microbial suppression in real-world storage conditions—an observation consistent with other studies on EO applications in fruits [33]. Interestingly, *A. radiobacter* remained the most resistant strain across all concentrations. This aligns with its ecological niche in the rhizosphere and lesser susceptibility to volatile antimicrobial agents compared to fruit pathogens. The mechanism of action is likely linked to the membrane-targeting activity of small volatile components (e.g., linalool, eugenol), which increase membrane fluidity and permeability, ultimately causing cell death. This mechanism is well documented in vapor-phase antimicrobial studies [32]. Moreover, studies on EOs in the minimal processing of fruits highlight the significance of volatility and application methods, showing that realistic vapor-phase treatments can effectively reduce the microbial load when applied properly [34].

A discrepancy was obtained in large inhibition zones, but high MIC values may reflect the different sensitivities of the agar diffusion and broth dilution methods, which are influenced by compound diffusion rates and solubility [29]. This supports its known susceptibility to plant-derived antimicrobials, particularly lipophilic compounds like terpenoids, which more easily penetrate Gram-positive bacterial membranes [35]. Conversely, *P. syringae* displayed the lowest MIC_90_ (5.17 mg/mL), suggesting a higher intrinsic sensitivity in broth cultures despite its limited inhibition zone in diffusion assays. Strains such as *P. carotovorum* and *X. arboricola* showed intermediate responses in both assays, with inhibition zones around 6.33–6.67 mm and MIC_90_ values between 5.86 and 6.89 mg/mL. These results are consistent with prior studies demonstrating that EOs containing sesquiterpenes and oxygenated monoterpenes can affect the permeability of Gram-negative bacteria, albeit at slightly higher concentrations than for Gram-positives [30,36]. The contrasting responses observed between the agar diffusion and MIC methods underscore the importance of using complementary techniques when evaluating the antimicrobial potential of EOs. While agar diffusion favors compounds with high volatility and good diffusion properties, the broth microdilution method provides a more accurate quantification of growth inhibition in liquid environments [29]. These findings suggest that ivy EO possesses a broad-spectrum antimicrobial activity, though its efficacy is moderate and requires relatively high concentrations to achieve strong inhibitory effects. A further chemical characterization of its major constituents, such as hederagenin derivatives or saponins, may help clarify the specific mechanisms of action.

The observed pattern of the in situ effect of ivy EO’s strongest inhibition at 500 µg/L and the clear decline in efficacy at lower concentrations corroborate the previous findings [30], which emphasized the critical roles of vapor pressure and concentration for EO components to maintain antimicrobial activity in food systems. The gradual increase in inhibition with concentration also supports the idea that ivy EO acts via a physicochemical mechanism in the vapor phase, most likely by disrupting membrane integrity through the diffusion of active compounds such as terpenoids and sesquiterpenes [35,36]. The most notable concentration effect was observed in *A. radiobacter* and *P. syringae*, with over 40 percentage points of difference in inhibition between 62.5 and 500 µg/L. This suggests that, in addition to their sensitivity, these strains may have nonlinear responses to vapor-phase EO exposure, possibly due to changes in membrane fluidity or stress adaptation thresholds [32,36]. Unlike in vitro conditions, where antimicrobial activity is often influenced by solubility and diffusion limitations, in situ vapor-phase treatments allow the uniform dispersion of volatile compounds across the fruit surface. This could explain the significantly higher efficacy of ivy EO in situ compared to its performance in direct-contact tests. Similar effects have been observed with other EOs in vapor form, where antimicrobial activity increased substantially when applied in enclosed systems [33].

EOs contain several compounds involved in the plant stress response. Notably important are tocopherols, especially α-tocopherol and γ-tocopherol, which is catalyzed into α-tocopherol by the enzyme γ-tocopherol methyltransferase [37]. These compounds are primarily known as antioxidants that help plants cope with abiotic stress. They also act as signaling molecules for phytohormone jasmonic acid [10], which directly induces the expression of *ypr10* genes [34,38]. Another group of EO components involved in plant stress responses are unsaturated fatty acids. α-linolenic acid (ALA) and linoleic acid (LA) serve as precursors to oxylipins, a class of signaling molecules that mediate plant stress responses. ALA is the initial substrate for the biosynthesis of jasmonic acid [39]. Terpenes such as linalool can also be mentioned, as they synergistically enhance the antimicrobial efficacy of EOs [40]. In addition, nerolidol exhibits both antibacterial and antioxidant activity [41]. Isoeugenol and eugenol are phenylpropanoids found in the receptacle and achenes of strawberries, present in concentrations ranging from 25 to 150 ng·g^−1^ dry weight depending on the developmental stage [42]. These compounds exhibit significant antimicrobial activity against fungi as well as a broad spectrum of Gram-positive and Gram-negative bacteria [43].

In the present study, an in silico molecular docking approach was employed to elucidate the potential antibacterial mechanisms of the volatile constituents of strawberry and ivy EOs against the key bacterial enzymes responsible for cell wall synthesis, nucleic acid replication, and protein biosynthesis. The computational findings were correlated with in vitro antibacterial assays conducted in this study.

Overall, the docking results revealed that compounds from ivy EO exhibited significantly higher binding affinities toward the selected bacterial proteins compared with those from strawberry EO, suggesting stronger molecular-level interactions. Among the tested ligands, *p*-cymene demonstrated the most favorable binding with D-alanine–D-alanine ligase (−7.9 kcal mol^−1^), an essential enzyme that catalyzes the ATP-dependent condensation of two D-alanine molecules to form the D-Ala–D-Ala dipeptide. This dipeptide constitutes the terminal motif of the peptidoglycan monomer, crucial for bacterial cell wall cross-linking and structural integrity [44]. The strong binding of *p*-cymene to this enzyme indicates a potential inhibition of peptidoglycan biosynthesis, leading to a compromised cell wall formation and bacterial lysis. This effect is particularly pronounced in Gram-positive bacteria such as *P. megaterium*, which depend heavily on a thick peptidoglycan layer for mechanical stability and protection.

In addition, *p*-cymene displayed substantial binding affinities toward DNA gyrase (−5.8 kcal mol^−1^), dihydropteroate synthase (−5.1 kcal mol^−1^), and penicillin-binding protein 1a (−6.0 kcal mol^−1^). These enzymes collectively regulate essential cellular processes such as DNA supercoiling during replication and transcription (DNA gyrase), tetrahydrofolate biosynthesis required for nucleotide formation (dihydropteroate synthase), and peptidoglycan cross-linking (penicillin-binding protein 1a) [45,46]. Although it is possible that PBP1a in its current form may not be present in all the bacteria, its functional homologs can be present, which are analogous to PBP1a. The inhibition of these enzymes can disrupt DNA replication, nucleotide metabolism, and cell wall remodeling, all of which are indispensable for bacterial survival. Since Gram-negative bacteria such as *P. carotovorum*, *P. syringae*, and *X. arboricola* possess an additional outer membrane that often restricts antimicrobial entry, the ability of ivy EO constituents to interact with both cell wall- and DNA-associated enzymes suggests their capacity to permeate or destabilize the outer membrane, contributing to their efficacy against these species.

Similarly, 1,8-cineole, the most abundant constituent of ivy EO, exhibited strong docking interactions with topoisomerase IV (−6.1 kcal mol^−1^) and dihydrofolate reductase (−6.0 kcal mol^−1^). Topoisomerase IV plays a pivotal role in decatenating replicated DNA molecules during cell division, while dihydrofolate reductase catalyzes the reduction of dihydrofolate to tetrahydrofolate, a key step in thymidylate biosynthesis essential for DNA replication and repair [46]. The inhibition of these enzymes can result in defective chromosome segregation and impaired DNA synthesis, suppressing bacterial proliferation across both Gram-positive and Gram-negative species.

When correlated with the antibacterial assay results, the stronger inhibitory potential of ivy EO can be attributed to the effective binding of *p*-cymene and 1,8-cineole to these vital enzymes. The relatively lower activity of strawberry EO corresponds with its weaker binding affinities (−3.8 to −6.1 kcal mol^−1^), suggesting a limited interaction with active site residues and thus a reduced inhibition efficiency. The pronounced activity of ivy EO against *P. megaterium* further supports the hypothesis that the inhibition of D-alanine–D-alanine ligase and PBP 1a compromises cell wall integrity, which is especially critical for Gram-positive bacteria due to their thick, peptidoglycan-rich structure. Collectively, these findings indicate that the superior antibacterial activity of ivy EO is primarily governed by the synergistic action of *p*-cymene and 1,8-cineole, which effectively target multiple bacterial enzymes involved in vital biosynthetic and replication pathways. This dual inhibition mechanism, disrupting both cell wall synthesis and DNA replication, likely underlies the enhanced and broad-spectrum antibacterial effects of ivy EO against both Gram-positive and Gram-negative bacteria observed in vitro.

## 4. Materials and Methods

### 4.1. Material and Cultivation

**Figure 3:**
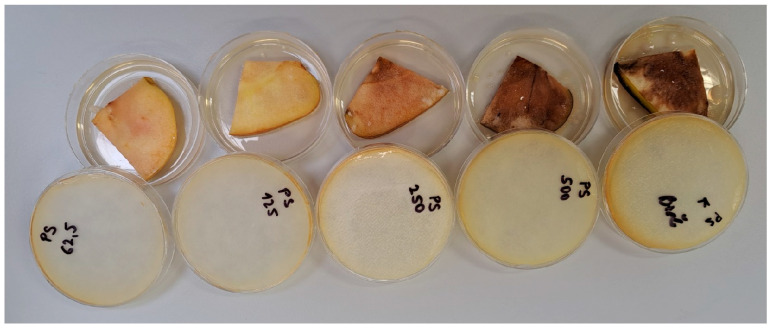
Application of *Pseudomonas syringae* to slices of the Smeralda apple variety plant model.

For the evaluation of antimicrobial activity, the Smeralda variety of *Malus domestica* Borkh. was used as a fruit model (Figure 3) and the following bacterial strains were selected: *Xanthomonas arboricola* CCM 1441, *Pectobacterium carotovorum* CCM 1008, *Pseudomonas syringae* CCM 2868, *Agrobacterium radiobacter* CCM 2926, and *Priestia* (*Bacillus*) *megaterium* CCM 2007. These strains were obtained from the Czech Collection of Microorganisms in Brno, Czech Republic, and were stored in a lyophilized state at −18 °C. For testing purposes, the lyophilized strains were rehydrated and cultured in Mueller–Hinton Broth (MHB, Oxoid, Basingstoke, UK) at 37 °C for 24 h, except for *Pseudomonas syringae*, which was cultured at 30 °C. After incubation, the bacterial cultures were adjusted to a 0.5 McFarland standard using a densitometer, corresponding to approximately 1.5 × 10^8^ colony-forming units (CFU) per mL. These standardized bacterial cultures were then prepared for antimicrobial activity testing.

### 4.2. Biological Material for Analyses

The biological material collection for gene expression analysis included three types of controls, five types of bacterial treatments, and two EO treatments, resulting in a total of seventeen samples. The three types of controls were a fresh apple (fresh control), an apple sample after seven days of cultivation (7th-day control), and five apple samples after seven days of cultivation, each treated with a different bacterial species (no-EO control). EOs were applied only in combination with bacterial inoculation and were interpreted relative to the corresponding “no-EO” bacterial controls.

The plant material was grounded in a pre-chilled mortar with sand and a pestle. The total RNA was extracted using the Ribospin™ Plant (GeneAll^®^, Seoul, Republic of Korea) kit following the original protocol, with a change in the lysis step (350 µL of each lysis buffer were combined). The quality and quantity of RNAs were checked using a NanoPhotometer^®^ P-360 (Implen GmbH, Munchen, Germany) at an absorbance ratio of A260/280. Samples with a good quantity but inadequate quality were purified using the GeneJET™ RNA Cleanup and Concentration Micro Kit (Thermo Scientific™, Abington, UK). The total amount of 350 ng RNA was transcribed using the Maxima First Strand cDNA Synthesis Kit for RT-qPCR (Thermo Scientific™, Abington, UK). The Minus-RT control was also transcribed with the use of all samples’ mixed RNA templates to ensure DNA absence.

### 4.3. Gene Expression Analysis

*MdACT* (NCBI ID DT002474) was used as a reference gene suitable for expression analysis in *Malus domestica*. The expression level of *MdPR10* for Mal d 1 was used to measure the ability of EOs to protect apples from biological stress induced by bacteria. Primer pair sequences were *MdACT* fwd 5′CTATGTTCCCTGTTTTTCATGC3′ and *MdACT* rev 5′GCCAACCTTGTTTTTCATGC3′ and *MdPR10* fwd 5′GATTGAAGGAGATGCTTTGACA3′ and *MdPR10* rev 5′TTGGTGTGGTAGTGGCTGATA3′, both designed by [47]. Each reaction mixture contained EliZyme™ Green MIX add ROX (Elisabeth Pharmacon^®^, Brno, Czech Republic), 0.08 µL 100X ROX, 1 µL cDNA diluted 1:4 (*v*/*v*), 300 nM (*MdACT*)/900 nM (*MdPR10*) of primers, and nuclease-free water. PCR conditions for *MdACT* were 95 °C for 2 min; 45 cycles of denaturation at 95 °C for 5 s; and annealing and elongation at 60 °C for 15 s. The annealing + elongation step was changed at 63 °C for 10 s for *MdPR10*. Each PCR was followed using a melting curve from 70 to 95 °C at 1 °C increments to clarify the specificity of the PCR products. All PCRs were performed in technical triplicates; negative water controls were checked in each run.

### 4.4. HS-GC/MS Analysis of EOs

The vapor-phase chemical composition of strawberry and ivy EOs was investigated using a Perkin-Elmer Headspace Turbomatrix 40 autosampler connected to a Perkin Elmer Clarus 500 gas chromatograph (Perkin Elmer, Waltham, MA, USA), coupled with a mass spectrometer and equipped with a flame ionization detector (FID). For the extraction of the volatiles, 1mL of each EO was placed into a 15 mL glass vial with a PTFE-coated silicone septum and the operative applied conditions following [48] with slight modifications. A Varian Factor Four VF-1 (60 m × 0.32 mm, 1.0 μm of film thickness) capillary column was used to separate the components. The chromatographic conditions were as follows: from 60 °C to 220 °C at a rate of 6 °C/min and held for 15 min. Helium was used as a gas carrier, with a flow rate of 1.0 mL min^−1^ and a split ratio of 20:1. The mass spectra were obtained in the electron impact mode (EI) at 70 eV in scan mode, ranging 35–550 *m*/*z*.

The volatile compounds were identified through the comparison of the MS-fragmentation patterns of the analytes with those of pure components stored in the Wiley 2.2 and Nist mass spectra library databases. Furthermore, the Linear Retention Indices (LRIs) were calculated using a series of *n*-alkane standards and compared with those reported in the literature (Nist 11). The relative amounts of the components were expressed as a percentage of the peak area to the total peak area, without the use of an internal standard or any factorial correction. The analyses were carried out in triplicate.

### 4.5. Bacterial Response Analysis

#### 4.5.1. Disk Diffusion Assay

For antimicrobial testing, 100 μL of bacterial suspension (prepared as described in Section 4.1) was pipetted onto Mueller–Hinton Agar (MHA, Oxoid, Basingstoke, UK). Sterile paper disks (6 mm diameter; Oxoid, Basingstoke, UK) were placed onto the surface of the inoculated agar. Each disk was loaded with 10 μL of EO. The plates were incubated for 24 h at 37 °C for most bacterial strains, and at 30 °C for *Pseudomonas syringae*. As positive controls, standard antibiotics (cefoxitin and gentamicin; Oxoid, Basingstoke, UK) were used—one for the Gram-negative and one for the Gram-positive strain. Negative controls consisted of disks impregnated with the EO solvent (ultrapure water) and blank disks without any treatment. The diameter (radius) of inhibition zones around each disk was measured in mL at three points, and the average and standard deviation were calculated. All measurements were performed in triplicate.

#### 4.5.2. Minimum Inhibitory Concentration (MIC) Determination

The minimum inhibitory concentrations (MIC_50_ and MIC_90_) of the tested EOs were determined though broth microdilution in sterile 96-well microplates. Each well was filled with 50 μL of standardized microbial suspension and 50 μL of serially diluted EO in Mueller–Hinton Broth (MHB) for bacteria or Sabouraud Dextrose Broth (SDB) for yeasts. The final concentrations of EOs ranged from 10 mg/mL to 0.00488 mg/mL. Negative controls contained a medium with EO but no microbial inoculum. Positive controls consisted of inoculated broth without EO. The plates were incubated for 24 h at 37 °C for bacterial strains and 25 °C for yeasts. Microbial growth was assessed spectrophotometrically at 570 nm using a Glomax microplate reader (Promega, Madison, WI, USA). MIC_50_ and MIC_90_ were defined as the lowest EO concentrations that inhibited microbial growth by at least 50% and 90%, respectively. All assays were performed in triplicate to ensure reproducibility. MIC values were statistically estimated using probit analysis.

#### 4.5.3. In Situ Antimicrobial Assay

To mimic real-life contamination conditions, selected Gram-positive and Gram-negative bacterial strains, along with yeast isolates, were applied to fresh apple slices as a natural food matrix. Commercial apples were first washed, then sliced into 0.5 mm sections, and air-dried under sterile conditions. The prepared slices were placed in sterile 60 mm Petri dishes and inoculated with standardized microbial suspensions. EOs were dissolved in ethyl acetate to obtain concentrations of 500, 250, 125, and 62.5 μg/L. Sterile filter paper disks were impregnated with the EO solutions and attached to the inner surface of the Petri dish lids, allowing exposure to EO vapors. Control plates contained disks treated with ethyl acetate only. All dishes were hermetically sealed and incubated at 37 °C for 7 days. After incubation, microbial growth on the apple surface was assessed using standard cultivation techniques. Growth inhibition was evaluated semi-quantitatively by analyzing images with ImageJ 1.54p software (NIH, Bethesda, MD, USA), providing insight into the vapor-phase antimicrobial efficacy of the EOs under in situ conditions. This in situ assay was designed to quantify vapor-phase antimicrobial efficacy under deliberate microbial challenge; therefore, sensory, nutritional, and storage-quality parameters were not evaluated within this inoculated model.

### 4.6. Molecular Docking

To elucidate the molecular basis of the antibacterial potential of essential oils (EOs), docking analyses were performed to evaluate the interactions between their bioactive constituents and key bacterial target proteins. In this regard, seven universal bacterial enzymes were selected based on their essential roles in cell survival and replication: isoleucyl-tRNA synthetase (PDB ID: 1JZQ), DNA gyrase (PDB ID: 1KZN), dihydropteroate synthase (PDB ID: 2VEG), D-alanine–D-alanine ligase (PDB ID: 2ZDQ), topoisomerase IV (PDB ID: 3RAE), dihydrofolate reductase (PDB ID: 3SRW), and penicillin-binding protein 1a (PDB ID: 3UDI) [46,49,50]. The crystal structures of these proteins were retrieved from the Protein Data Bank (https://www.rcsb.org/, accessed on 10 October 2025).

Major EO components, identified through GC–MS analysis with relative abundances exceeding 1%, were obtained from the PubChem database in 3D SDF format (https://pubchem.ncbi.nlm.nih.gov/, accessed on 11 October 2025). Ligands were energy-minimized and geometry-optimized, followed by a conversion to PDBQT format for compatibility with docking. Molecular docking was performed using PyRx following the protocol described by [50]. The binding affinities were computed to identify the most favorable ligand–protein interactions. Top-ranked docking poses, determined using binding energy scores, were further analyzed and visualized using PyMOL (v3.0.4) and Discovery Studio Biovia (v24.1.0.23298).

### 4.7. Data Analysis

Relative gene expression levels were calculated using the ΔΔCt method [51] written for the Microsoft Excel platform and expressed as fold changes relative to the relevant controls. This method implements the effectivity of PCRs into calculating the resulting fold changes. The calculations were as follows: the 7th-day control was compared to the fresh control. The fold change of the no-EO control was calculated by comparing to the *7th-day control*. The fold change of the other samples (with bacteria and EO) were compared to the no-EO control. A single-factor ANOVA was also calculated in Excel using the DataAnalysis package. For the microbial analysis, ANOVA was applied, followed by a post hoc Tukey’s test using the online, freely available ANOVA Calculator URL www.statskingdom.com (accessed on 15 March 2025).

## 5. Conclusions

Overall, it can be concluded that strawberry EO is the most effective against non-pathogenic or weakly virulent strains (notably *P. megaterium* and *A. radiobacter*), while more aggressive pathogens such as *P. carotovorum* and *P. syringae* were significantly more resistant to its effects. The determined MIC values suggest that, for practical applications in plant protection, it would be necessary to optimize the dosing and/or combine strawberry EO with synergistic bioactives. Strawberry EO demonstrated a higher antimicrobial efficacy in vitro, particularly against *P. megaterium* and *A. radiobacter*, indicating its potential under controlled laboratory conditions.

However, the in situ experiments revealed that ivy EO exerted significantly greater inhibitory effects, suppressing the growth of all tested phytopathogenic bacteria directly on apple tissues. These results suggest that ivy EO may offer more practical value for preserving fresh produce under realistic, postharvest storage conditions. Our findings further underscore the potential of ivy EO as a natural preservative for fresh produce, particularly under postharvest storage conditions where controlled atmospheres can enhance the action of volatile bioactive substances. The high inhibition rates across both Gram-positive and Gram-negative bacteria suggest that ivy EO may be suitable for use in integrated antimicrobial strategies, such as active packaging and minimally processed fruit preservation.

## Figures and Tables

**Table 1:** Volatile chemical composition (percentage mean value ± standard deviation) of *strawberry* and *ivy* EOs.

N°	Component ^1^	LRI ^2^	LRI ^3^	EO ^4^	EO ^5^
1	Ethylbutyrate	802	800	32.2 ± 0.02	-
2	Ethyl 2-methylbutanoate	844	846	30.6 ± 0.02	-
3	Ethyl-3-methylbutanoate	852	849	11.6 ± 0.02	-
4	Cis-3-exen-1-ol	857	855	0.9 ± 0.02	-
5	α-pinene	928	932	0.8 ± 0.02	8.2 ± 0.02
6	Camphene	936	942	-	1.2 ± 0.02
7	Sabinene	968	972	-	2.4 ± 0.02
8	β-pinene	982	981	0.3 ± 0.02	-
9	Ethyl hexanoate	1015	1011	12.3 ± 0.02	13.5 ± 0.02
10	3-carene	107	1014	-	8.3 ± 0.02
11	Hexyl acetate	1022	1018	4.8 ± 0.02	-
12	*p*-cymene	1024	1022	-	5.2 ± 0.02
13	Limonene	1033	1029	0.1 ± 0.02	-
14	1,8-cineole	1036	1033	0.6 ± 0.02	47.5 ± 0.02
15	γ-terpinene	1065	1062	-	8.8 ± 0.02
16	Terpinolene	1092	1097	-	2.4 ± 0.02
17	Thujone	1098	1099	-	0.5 ± 0.02
18	Isopentyl isovalerate	1105	1100	4.1 ± 0.02	-
19	2-methylbutyl-isovalerate	1108	1107	1.6 ± 0.02	-
20	Camphor	1148	1151	-	tr
21	Terpinen-4-ol	1179	1182	-	0.8 ± 0.02
22	Linalool formate	1210	1206	-	0.2 ± 0.02
23	Carvone	1231	1226	0.1 ± 0.02	-
24	Borneol formate	1235	1228	-	0.5 ± 0.02
25	β-caryophyllene	1427	1435	-	0.2 ± 0.02
26	Aromadendrene	1455	1460	-	0.1 ± 0.02
	SUM			100.0	99.8
	Monoterpenes			1.2	50.0
	Monoterpene alcohol			-	0.8
	Monoterpenoids			0.7	48.7
	Sesquiterpenes			-	0.2
	Fatty acid ester			97.2	-
	Others			0.9	0.1

^1^ The components are reported according to their elution order on apolar column; ^2^ Linear Retention Indices measured on apolar column; ^3^ Linear Retention Indices from the literature; ^4^ volatile chemical composition of strawberry EO; ^5^ volatile chemical composition of ivy EO; tr: percentage mean values ≤ 0.1%; -: not detected.

**Table 2:** In vitro antimicrobial activity for strawberry essential oil (zone of inhibition in mm).

Strawberry Essential Oil		
Bacteria	Inhibition Zone	ATB
*Agrobacterium radiobacter* CCM 2926	5.33 ± 0.58 ^a^	29.67 ± 0.58 ^a^
*Priestia* (*Bacillus*) *megaterium* CCM 2007	7.33 ± 0.58 ^c^	32.33 ± 0.58 ^a^
*Pectobacterium carotovorum* CCM 1008	3.33 ± 0.58 ^b^	33.33 ± 0.58 ^a^
*Pseudomonas syringae* CCM 2868	2.33 ± 0.58 ^b^	31.33 ± 0.58 ^a^
*Xanthomonas arboricola* CCM 1441	5.67 ± 0.58 ^a^	30.33 ± 0.58 ^a^

Same upper index letters in the row show statistically significant differences among the groups.

**Table 3:** Minimal inhibitory concentration in mg/mL for strawberry essential oil.

Strawberry Essential Oil		
Bacteria	MIC_50_	MIC_90_
*Agrobacterium radiobacter* CCM 2926	3.28 ± 0.25 ^a^	3.45 ± 0.12 ^a^
*Priestia* (*Bacillus*) *megaterium* CCM 2007	3.37 ± 0.90 ^a^	3.51 ± 1.09 ^a^
*Pectobacterium carotovorum* CCM 1008	5.16 ± 1.11 ^a^	5.73 ± 0.91 ^b^
*Pseudomonas syringae* CCM 2868	5.34 ± 2.26 ^a^	7.01 ± 0.10 ^c^
*Xanthomonas arboricola* CCM 1441	4.33 ± 0.98 ^a^	4.99 ± 0.85 ^ab^

Same upper index letters in the row show statistically significant differences among the groups.

**Table 4:** In situ antimicrobial effect of strawberry essential oil on apple model.

Strawberry Essential Oil				
Bacteria	Concentration of PMEO in μg/L			
	62.5	125	250	500
*Agrobacterium radiobacter* CCM 2926	11.28 ± 1.07 ^a^	24.66 ± 2.40 ^a^	31.64 ± 1.54 ^a^	46.69 ± 2.81 ^a^
*Priestia* (*Bacillus*) *megaterium* CCM 2007	11.35 ± 1.26 ^a^	24.88 ± 1.98 ^a^	30.17 ± 1.18 ^a^	46.96 ± 1.53 ^a^
*Pectobacterium carotovorum* CCM 1008	13.31 ± 0.23 ^a^	22.80 ± 0.42 ^a^	31.92 ± 2.39 ^a^	45.23 ± 1.61 ^a^
*Pseudomonas syringae* CCM 2868	12.21 ± 0.86 ^a^	25.76 ± 1.70 ^a^	31.4 ± 0.65 ^a^	45.66 ± 2.15 ^a^
*Xanthomonas arboricola* CCM 1441	12.99 ± 0.53 ^a^	24.22 ± 1.65 ^a^	31.60 ± 1.24 ^a^	46.42 ± 2.24 ^a^

Same upper index letters in the row show statistically significant differences among the groups.

**Table 5:** In vitro antimicrobial activity for ivy essential oil (zone of inhibition in mm).

Strawberry Essential Oil		
Bacteria	Inhibition Zone	ATB
*Agrobacterium radiobacter* CCM 2926	11.67 ± 0.58 ^a^	0.00 ± 0.00 ^a^
*Priestia* (*Bacillus*) *megaterium* CCM 2007	8.33 ± 0.58 ^b^	0.00 ± 0.00 ^a^
*Pectobacterium carotovorum* CCM 1008	6.67 ± 0.58 ^c^	23.33 ± 0.58 ^b^
*Pseudomonas syringae* CCM 2868	6.33 ± 0.58 ^c^	12.33 ± 0.58 ^c^
*Xanthomonas arboricola* CCM 1441	6.33 ± 0.58 ^c^	9.33 ± 0.58 ^d^

Same upper index letters in the row show statistically significant differences among the groups.

**Table 6:** Minimal inhibitory concentration in mg/mL for ivy essential oil.

Strawberry Essential Oil		
Bacteria	MIC_50_	MIC_90_
*Agrobacterium radiobacter* CCM 2926	6.99 ± 1.05 ^a^	7.52 ± 0.99 ^a^
*Priestia* (*Bacillus*) *megaterium* CCM 2007	4.73 ± 0.83 ^b^	6.39 ± 0.01 ^ab^
*Pectobacterium carotovorum* CCM 1008	5.38 ± 0.67 ^ab^	5.86 ± 0.57 ^b^
*Pseudomonas syringae* CCM 2868	4.96 ± 0.63 ^ab^	5.17 ± 0.01 ^b^
*Xanthomonas arboricola* CCM 1441	6.23 ± 0.75 ^ab^	6.89 ± 0.52 ^ab^

Same upper index letters in the row show statistically significant differences among the groups.

**Table 7:** In situ antimicrobial effect of ivy essential oil on apple model.

Strawberry Essential Oil				
Bacteria	Concentration of PMEO in μg/L			
	62.5	125	250	500
*Agrobacterium radiobacter* CCM 2926	46.36 ± 1.59 ^a^	56.50 ± 0.30 ^ab^	66.5 ± 2.08 ^a^	87.86 ± 0.52 ^a^
*Priestia* (*Bacillus*) *megaterium* CCM 2007	47.71 ± 0.44 ^a^	56.95 ± 1.78 ^a^	67.25 ± 1.97 ^a^	87.57 ± 0.81 ^a^
*Pectobacterium carotovorum* CCM 1008	47.56 ± 1.36 ^a^	53.28 ± 1.05 ^b^	65.99 ± 0.43 ^a^	87.57 ± 0.81 ^a^
*Pseudomonas syringae* CCM 2868	45.08 ± 2.6 ^a^	53.94 ± 1.27 ^ab^	65.93 ± 2.88 ^a^	86.89 ± 1.13 ^a^
*Xanthomonas arboricola* CCM 1441	44.98 ± 2.07 ^a^	55.96 ± 1.68 ^ab^	68.50 ± 1.04 ^a^	84.83 ± 0.77 ^b^

Same upper index letters in the row show statistically significant differences among the groups.

**Table 8:** Binding free energy values (ΔG in kcal/mol) of volatile compounds of strawberry and ivy EOs with selected bacterial proteins.

Compounds		Binding Free Energy (ΔG in kcal/mol)						
EO4/Strawberry	CID	1JZQ	1KZN	2VEG	2ZDQ	3RAE	3SRW	3UDI
**Ethylbutyrate**	7762	−4	−4	−3.8	−4.3	−4	−4.1	−4.2
**Ethyl 2-methylbutanoate**	24,020	−4.1	−4.2	−3.9	−4.8	−4.3	−4.4	−4.6
**Ethyl-3-methylbutanoate**	7945	−4.3	−4.4	−4.2	−4.7	−3.9	−4.5	−4.6
**Ethyl hexanoate**	31,265	−4.3	−4.4	−4.1	−5.3	−4.4	−4.8	−4.4
**Hexyl acetate**	8908	−4.6	−4.7	−3.9	−4.7	−4.2	−4.8	−4.4
**Isopentyl isovalerate**	12,613	−4.9	−5.2	−4.2	−6	−4.5	−5.3	−4.8
**2-methylbutyl-isovalerate**	62,445	−5.2	−5.1	−4.5	−6.1	−5	−5.2	−5
**EO5/Ivy EOs**								
***α*** **-pinene**	6654	−5.8	−4.7	−4.5	−6.2	−5.1	−5.6	−5.4
**Camphene**	6616	−6.1	−4.8	−4.6	−5.8	−5	−5.5	−5.5
**Sabinene**	18,818	−5.8	−5.6	−4.9	−6.6	−6	−5.6	−6
**Ethyl hexanoate**	31,265	−4.3	−4.4	−4.1	−5.3	−4.4	−4.8	−4.4
**3-carene**	26,049	−6	−4.9	−4.9	−6.7	−5.7	−5.8	−6.2
***p*** **-cymene**	7463	−5.8	−5.8	−5.1	−7.9	−5.7	5.7	−6
**1,8-cineole**	2758	−5.9	−4.6	−4.5	−6.3	−6.1	−6	−5.4
***γ*** **-terpinene**	7461	−5.9	−5.5	−5	−7.2	−6.1	−5.6	−5.6
**Terpinolene**	11,463	−6	−5.8	−5	−7.1	−5.8	−5.6	−6

Protein PDB ID: 1JZQ—isoleucyl-tRNA synthetase, 1KZN—DNA gyrase, 2VEG—dihydropteroate synthase, 2ZDQ—d-alanine:d-alanine ligase, 3RAE—topoisomerase 4, 3SRW—dihydrofolate reductase, and 3UDI—penicillin-binding protein.

## Data Availability

The original contributions presented in this study are included in the article. Further inquiries can be directed to the corresponding author.

## Abbreviations

The following abbreviations are used in this manuscript:

ATB	Antimicrobial activity
Ct	Threshold cycle
EO	Essential oil
*MdPR10*	Gene for pathogenesis-related protein of *Malus domestica*
MIC	Minimal inhibition concentration
PCR	Polymerase chain reaction
